# Surfactants Significantly Improved the Oral Bioavailability of Curcumin Amorphous Solid Dispersions and Its Underlying Mechanism

**DOI:** 10.3390/pharmaceutics17121541

**Published:** 2025-11-29

**Authors:** Jinhua Yuan, Siyi Mao, Xiuzhen Ma, Xiaoling Liu, Yuejie Chen

**Affiliations:** 1 School of Pharmacy, Minzu University of China, Beijing 100081, China; 23302010@muc.edu.cn (J.Y.); 24302083@muc.edu.cn (S.M.); 25302185@muc.edu.cn (X.M.); 2 School of Life Sciences, Beijing University of Chinese Medicine, Beijing 102401, China; 3 Key Laboratory of Mass Spectrometry Imaging and Metabolomics, Minzu University of China, State Ethnic Affairs Commission, Beijing 100081, China

**Keywords:** curcumin, surfactant, bioavailability, amorphous solid dispersion (ASD), drug–polymer-surfactant interactions, dissolution enhancement

## Abstract

**Background/Objectives:** Surfactants are commonly used in amorphous solid dispersions (ASDs) to improve drug dissolution. A mechanistic understanding of their impact on in vitro dissolution and in vivo pharmacokinetics is essential for rational ASD design and for establishing predictive in vitro–in vivo correlation (IVIVC). **Methods:** Binary (Cur/P188) and ternary (Cur/P188/TW80, Cur/P188/SLS) ASDs were prepared by rotary evaporation. Drug–polymer–surfactant interactions were characterized by ^1^H NMR and FT-IR spectroscopy. To elucidate the bioavailability enhancement mechanism, we performed (i) in vitro non-sink dissolution to assess dissolution kinetics, nanostructure formation, and precipitate transformation; (ii) cellular uptake assays; and (iii) in vivo pharmacokinetic studies. **Results:** Cur self-associates via hydrogen bonding and π-π stacking, limiting its solubility. Polymer carrier P188 disrupts these interactions and forms stronger drug–polymer bonding. Surfactants TW80 and SLS exhibited distinct interaction profiles: TW80 competitively disrupted Cur-P188 bonding, whereas SLS integrated into the Cur-P188 assembly to form stable ternary nanostructures. The Cur/P188/SLS ASD achieved the highest and most sustained supersaturation, maintained amorphous precipitates, and enhanced cellular uptake, leading to significantly improved oral bioavailability. **Conclusions:** Surfactants critically influence ASD performance by preserving high-energy drug states through three key mechanisms: (1) generating and maintaining supersaturation, (2) facilitating nanostructure formation, and (3) stabilizing amorphous precipitates. These mechanisms collectively enhance cellular uptake and bioavailability. Our findings demonstrate that both dissolution and in vivo performance are governed by multifaceted drug–polymer–surfactant interactions, providing critical insights into surfactant functionality and IVIVC to guide rational ASD formulation.

## 1. Introduction

Poorly water-soluble drugs typically exhibit low oral bioavailability due to poor absorption in the gastrointestinal (GI) tract [1]. These properties may compromise therapeutic efficacy and even cause safety issues [2]. Various techniques have been developed to improve the solubility and absorption of such drugs, including micronization, complexation, salt formation, nanonization, and supersaturated drug delivery systems [3]. Among these, amorphous solid dispersion (ASD) has attracted significant attention and has proven to be highly promising. In ASD formulations, the drug is molecularly dispersed within a polymer matrix, losing its crystalline structure to exhibit an amorphous nature, which improves wettability and solubility [4]. ASDs represent a simple and reliable technique suitable for a wide range of chemically diverse drugs. However, ASD development is often challenged by issues such as poor drug–polymer miscibility, inherent hydrophobicity, and limited physical stability [5].

The incorporation of surfactants into ASDs provides an effective means to overcome these limitations [6]. Owing to their amphiphilic nature, surfactants significantly improve formulation wettability, thereby enhancing the dissolution and ultimately improving their bioavailability [7]. However, the incorporation of surfactants may also bring some risks, such as reducing the physical stability of ASDs during manufacturing or storage, as well as promoting drug precipitation during dissolution. These effects can ultimately compromise the in vitro and in vivo performance of the ASD [8]. Despite their widespread application, the influence of surfactants on key ASD properties, including crystallization inhibition, dissolution behavior, and bioavailability remains inadequately understood.

The impact of surfactants on the key pharmaceutical properties of ASDs is complex and multifaceted. Effective surfactants promote the formation of homogeneous ASDs by improving drug–polymer miscibility during preparation. A uniform solid-state dispersion facilitates intimate drug–polymer contact and interactions, thereby enhancing physical stability and dissolution performance [9]. By acting as plasticizers and/or improving wetting, surfactants facilitate drug release, thereby generating drug supersaturation levels exceeding the crystalline solubility, which is a critical factor for improving gastrointestinal absorption [10]. Simultaneously, surfactants help sustain supersaturation through micellar encapsulation or by modulating drug–polymer phase separation via liquid–liquid phase separation, resulting in the formation of drug-rich aggregates (LLPS-DAs) or nanodroplets that are critical for maintaining supersaturation. These high-energy nanodroplets or aggregates serve as reservoirs that maintain high free drug concentration in solution [11]. Furthermore, by improving dissolution and stabilizing the phase behavior of supersaturated solutions, surfactants can enhance membrane transport and ultimately improve oral absorption [12].

However, the incorporation of surfactants in ASDs does not invariably lead to improved dissolution, absorption, or bioavailability. In some cases, it may even produce the completely opposite effect. For example, surfactants can impair drug–polymer miscibility through strong interactions with the polymer matrix [13]. Their plasticizing effects may increase molecular mobility, accelerating phase separation or even inducing crystallization of the amorphous drugs [14]. Although surfactants promote drug release and supersaturation during dissolution, the resulting supersaturated state is thermodynamically unstable and prone to precipitation and crystallization, thereby negating the benefits of the amorphous form. Certain surfactants, such as sodium lauryl sulfate (SLS), may competitively disrupt drug–polymer interactions, weakening the polymer’s capacity to inhibit crystallization and ultimately reducing bioavailability [15]. Furthermore, the use of surfactant at insufficient concentrations, as demonstrated in a study where Poloxamer 188 was added below its critical micelle concentration to an l-THP ASD can induce precipitation during dissolution and reduce bioavailability [16].

Despite extensive research on surfactants in ASDs, a critical knowledge gap remains in understanding how specific surfactant chemistries mechanistically influence in vivo bioavailability. Previous studies have often focused on isolated aspects of surfactant functionality, such as solid-state stability or dissolution performance, without establishing an integrated view connecting molecular-level interactions to in vitro dissolution behavior and ultimately in vivo outcomes. In particular, the differential effects of surfactant type on the interrelationships among solid-state properties, solution behavior, transmembrane transport, and oral absorption, have not been systematically investigated. Consequently, current surfactant selection remains largely empirical, lacking a rational framework grounded in mechanistic insight and predictive capability.

This study aims to establish a mechanistic framework for rational surfactant selection in ASDs by systematically investigating how surfactant chemistry dictates formulation performance from molecular to physiological levels. Curcumin (Cur), a BCS Class IV polyphenol with poor aqueous solubility (~11 ng/mL) [17], high lipophilicity (logP ≈ 3) [18], and low oral bioavailability, was selected as the model drug. Poloxamer 188 (P188) served as the polymer carrier due to its established pharmaceutical utility. Two surfactants, polysorbate 80 (TW80) and SLS, were selected for comparative analysis based on their widespread pharmaceutical use and fundamentally distinct physicochemical properties. TW80 is a non-ionic surfactant known for its steric stabilization effects, whereas SLS is a strong anionic surfactant that exhibits high solubilizing capacity via micellization. We hypothesize that the distinct molecular architectures of TW80 and SLS differentially govern drug–polymer interactions, supersaturation behavior, and ultimately the oral bioavailability of Cur from Poloxamer 188-based ASD. To test this hypothesis, we employed a multi-modal analytical approach encompassing (1) solution-state ^1^H NMR and solid-state FT-IR spectroscopy to investigate drug–polymer–surfactant intermolecular interactions; (2) in vitro non-sink dissolution studies to evaluate supersaturation kinetics, nanostructure formation, and precipitate crystallization; (3) Madin-Darby Canine Kidney (MDCK) cell-based transport studies to evaluate drug uptake; and (4) in vivo pharmacokinetic studies to determine bioavailability. Through these correlated analyses, this work provides a comprehensive understanding that links molecular interactions to macroscopic dissolution behavior and ultimate bioavailability, offering a predictive strategy for surfactant optimization in ASD development.

## 2. Materials and Methods

### 2.1. Materials

Curcumin (Cur), polysorbate 80 (TW80), sodium lauryl sulfate (SLS), carboxymethyl cellulose sodium (CMC-Na), formic acid, and diclofenac sodium were sourced from Macklin Biochemical Co., Ltd. (Shanghai, China). Poloxamer 188 (P188) was kindly supplied by BASF Chemical Company Ltd. (Ludwigshafen, Germany). Ethanol (AR, 99%), methanol (LC-MS/MS-grade, ≥99.9%), ethyl acetate (LC-MS/MS-grade, ≥99.9%), acetonitrile (LC-MS/MS-grade, ≥99.9%), and deuterated chloroform (CDCl_3_) were obtained from Energy Chemical Company (Shanghai, China). Dimethyl sulfoxide (DMSO) was purchased from Sigma-Aldrich (St. Louis, MO, USA). Penicillin–streptomycin was acquired from Gibco (Grand Island, NY, USA). Madin–Darby Canine Kidney (MDCK, CVCL_0422) cells, fetal bovine serum (FBS), and the Cell Counting Kit-8 (CCK-8) were procured from Immocell Biotechnology Co., Ltd. (Xiamen, China).

**Figure 1:**
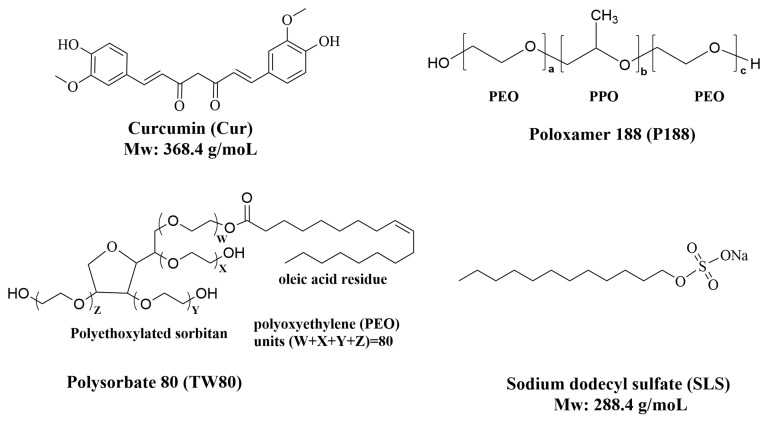
Chemical structures of the key components used in the formulation: Curcumin (Cur, active pharmaceutical ingredient), Poloxamer 188 (P188, polymeric carrier), polysorbate 80 (TW80, surfactant), and sodium lauryl sulfate (SLS, surfactant).

The chemical structures of the active pharmaceutical ingredient (Cur), the polymeric carrier (P188), and the surfactants (TW80 and SLS) are presented in Figure 1.

### 2.2. Methods

#### 2.2.1. Preparation of Cur ASDs

Cur ASDs were prepared at two different drug-to-excipient ratios to meet specific experimental requirements.

(1) Low drug-loading ASDs, including binary surfactant-free Cur/P188 ASD (1:8, *w*/*w*; 11 wt.% drug) and ternary surfactant-containing Cur/P188/TW80 and Cur/P188/SLS ASDs (1:8:1, *w*/*w*/*w*; 10 wt.% drug), were designed with higher polymer content to enhance dissolution performance and prolong supersaturation. These formulations were used for in vitro dissolution (Section 2.2.4), cellular uptake (Section 2.2.5), and in vivo pharmacokinetic studies (Section 2.2.6).
(2) High drug-loading ASDs, including Cur/P188 ASD (1:2, *w*/*w*; 33 wt.% drug) and Cur/P188/TW80 and Cur/P188/SLS ASDs (1:2:1, *w*/*w*/*w*; 25 wt.% drug), contained less polymer to maintain the amorphous state of the drug while minimizing spectral interference from the polymer. These formulations were specifically used for FT-IR studies (Fourier Transform Infrared Spectroscopy (FT-IR) section).

Pure amorphous Cur and all Cur ASDs were prepared by rotary evaporation. Precisely weighed Cur, P188, and surfactant (if applicable) were dissolved in ethanol to form a clear solution with a concentration of 20 mg/mL (corresponding to a 2% *w*/*v* solvent-to-solid ratio). The solvent was evaporated using a rotary evaporator at 60 °C under a reduced pressure of 20 mbar. The resulting solid was vacuum-dried (24 h) and stored at -20 °C for further use. The amorphous nature of Cur ASDs was confirmed by powder X-ray diffraction (PXRD, Powder X-Ray Diffraction (PXRD) section) before the experiment.

#### 2.2.2. Particle Characterization Methods

##### Powder X-Ray Diffraction (PXRD)

PXRD was used to characterize the crystalline nature of both freshly prepared Cur ASDs and precipitates collected during their dissolution. Measurements were conducted on a Rigaku Smartlab diffractometer (Rigaku Corporation, Tokyo, Japan) configured in Bragg–Brentano geometry using Cu Kα_1_ radiation. Diffractograms were collected over a 2θ range of 0° to 60° with a step size of 0.01°.

##### Dynamic Light Scattering (DLS)

DLS was used to characterize subvisible drug species formed during the dissolution of Cur ASDs. The hydrodynamic diameter and zeta potential of Cur nanostructures were determined using a Zetasizer Pro instrument (Malvern Panalytical, Malvern, UK) equipped for both DLS and electrophoretic light scattering (ELS). Samples were filtered (0.22 µm) to remove large aggregates prior to analysis. Each sample was measured in triplicate (n = 3), with 30 runs per measurement. Data were processed using the ZS Xplorer software version 3.3.0.42 suite.

##### Transmission Electron Microscopy (TEM)

TEM was further utilized to examine the morphology of subvisible drug species generated during ASD dissolution. Observations were carried out on an FEI Tecnai F20 microscope (Thermo Fisher Scientific, Hillsboro, OR, USA) operated at 200 kV. Samples were negatively stained with phosphotungstic acid before imaging.

#### 2.2.3. Characterization of Intermolecular Interactions

##### Nuclear Magnetic Resonance (NMR)

^1^H-NMR was employed to investigate solution-state interactions between the drug, polymer, and surfactant. The following samples were analyzed: pure Cur (5, 10, and 20 mg/mL); binary mixtures (1:1, *w*/*w*) of Cur/P188, as well as ternary mixtures (1:1:1, *w*/*w*/*w*) of Cur/P188/TW80 and Cur/P188/SLS (each component at 5 mg/mL). ^1^H-NMR spectra were acquired using a Bruker AVANCE III HD 500 MHz spectrometer (Bruker Corporation, Billerica, MA, USA). Deuterated chloroform (CDCl_3_), which does not interfere with molecular interactions between the components, was used as the solvent. Tetramethylsilane (TMS) was applied as an internal reference.

##### Fourier Transform Infrared Spectroscopy (FT-IR)

FT-IR spectroscopy was used to study solid-state interactions between the drug, polymer, and surfactant. The following samples were analyzed: pure amorphous Cur, binary Cur/P188 ASD (1:2, *w*/*w*), as well as ternary Cur/P188/TW80 and Cur/P188/SLS ASDs (1:2:1, *w*/*w*/*w*). All samples were vacuum-dried (>24 h) before measurement. Spectra were collected on a Bruker Vertex 70 spectrometer (Bruker Optik GmbH, Ettlingen, Germany) using an attenuated total reflectance (ATR) accessory in the mid-IR region (600–4000 cm^−1^) with 128 scans at a resolution of 4 cm^−1^.

#### 2.2.4. In Vitro Dissolution Studies

##### Solubility Measurement

The equilibrium solubility of crystalline Cur was measured in the following aqueous solutions: pure water, polymer P188 (8 mg/mL), surfactant TW80 or SLS (1 mg/mL), and their combinations (P188/TW80 or P188/SLS; 8:1 *w*/*w*) with P188 at 8 mg/mL and surfactant at 1 mg/mL in water (pH 7.0). Excess crystalline Cur was suspended in each solution, vortexed (1 min), sonicated (10 min), and incubated (37 °C, 24 h). After incubation, suspensions were filtered (0.22 μm), and Cur concentration in the filtrate was quantified using HPLC/UV-*Vis* (HPLC/UV-*Vis* Method section). All experiments were performed in quadruplicate (n = 4).

##### Non-Sink Dissolution

The dissolution behavior of Cur formulations was evaluated under *non-sink* conditions. Tested formulations included pure amorphous Cur, binary Cur/P188 ASD (1:8, *w*/*w*), and ternary Cur/P188/TW80 and Cur/P188/SLS ASDs (1:8:1, *w*/*w*/*w*). Each sample was introduced into 6 mL of water (pH 7.0) to achieve a Cur-equivalent concentration of 1 mg/mL at 37 °C. Aliquots (0.4 mL) were collected at 10, 20, and 45 min, as well as at 1h and 2 h, and immediately centrifuged (10,000 rpm, 3 min, 25 °C) to separate the supernatant from the precipitate. (1) The supernatant was diluted 1:2 (*v*/*v*) with methanol and analyzed for Cur concentration via HPLC/UV-*Vis*; (2) The precipitate was vacuum-dried and analyzed for crystallinity using PXRD.

In a separate experimental run, an aliquot collected at 10 min was centrifuged under identical conditions, and the resulting supernatant was filtered through a 0.22 μm membrane. The resulting filtrate was analyzed for nanoparticle size distribution by DLS and for morphology by TEM.

All dissolution experiments were conducted in triplicate (n = 3).

#### 2.2.5. Quantification of Cellular Uptake of Cur ASDs

##### Cytotoxicity Assay

Madin-Darby Canine Kidney (MDCK) cells were seeded in 96-well plates (1 × 10^5^ cells/well) and cultured for 24 h at 37 °C in complete DMEM (10% FBS, 1% penicillin–streptomycin) under 5% CO_2_. Cells were treated for 4 h with 100 µL of complete DMEM media (pH 7.4) containing crystalline Cur, amorphous Cur, binary Cur/P188 ASD (1:8, *w*/*w*), or ternary Cur/P188/TW80 or Cur/P188/SLS ASDs (1:8:1, *w*/*w*/*w*), at Cur-equivalent concentrations of 0 (vehicle), 2, 10, 25, 50, 100, and 200 μg/mL. After treatment, cells were washed three times with PBS (pH 7.4). Cell viability was assessed using a CCK-8 assay: 100 µL of serum-free DMEM and 10 µL of CCK-8 reagent were added to each well, followed by incubation for 2 h. Absorbance was measured at 450 nm using a Multiskan GO microplate reader (Thermo Fisher Scientific, Vantaa, Finland).

Cell viability was calculated according to the following equation:

Viability (%) = [(As − Aᵦ)/(Ac − Aᵦ)] × 100%

where

- A_s_ is the absorbance of experimental wells (cells + treatment + CCK-8);
- A_c_ is the absorbance of control wells (cells + medium + CCK-8, no drug);
- A_ᵦ_ is the absorbance of blank wells (medium + CCK-8, no cells, no drug).

All experiments were performed in quintuplicate (n = 5).

##### Cellular Uptake Study

MDCK cells were seeded in 24-well plates and cultured following the procedures described in Cytotoxicity Assay section. The cells were treated with 500 µL of complete DMEM medium (pH 7.4) containing crystalline Cur, amorphous Cur, binary Cur/P188 ASD (1:8, *w*/*w*), or ternary Cur/P188/TW80 or Cur/P188/SLS ASDs (1:8:1, *w*/*w*/*w*), at Cur-equivalent dose of 5 µg per well. After 10 min of incubation, the treatment was terminated by immediate removal of the medium. The cells were then washed three times with ice-cold PBS, lysed with 200 µL of 0.1% SDS (assisted by sonication), and dissolved in methanol at a 1:2 (*v*/*v*) ratio. The total intracellular Cur content was quantified using LC-MS/MS. All experiments were performed in quadruplicate (n = 4).

#### 2.2.6. In Vivo Pharmacokinetics Study of Cur ASDs

##### Animals

Male Sprague-Dawley (SD) rats (200 ± 20 g) were supplied by SPF Biotechnology Co., Ltd. (Beijing, China) and housed under standard laboratory conditions throughout the study. All animals were housed in a controlled environment (22 ± 2 °C, 55 ± 10% humidity) under a 12/12 h light/dark cycle, with free access to a standard laboratory rodent diet and water. A 12 h fasting period was implemented prior to the experiment, with water permitted ad libitum. All animal procedures were performed in strict accordance with protocols approved by the Biological and Medical Ethics Committee of Minzu University of China.

##### Pharmacokinetic Study

After fasting for 12 h (with free access to water), SD rats (n = 25) were randomly divided into five groups (n = 5 per group). Group 1: crystalline Cur (i.v., dissolved in saline with 5% DMSO). Group 2: crystalline Cur (p.o., suspended in 0.5% CMC-Na). Group 3: Cur/P188 ASD (1:8, *w*/*w*; p.o.). Group 4: Cur/P188/TW80 ASD (1:8:1, *w*/*w*/*w*; p.o.). Group 5: Cur/P188/SLS ASD (1:8:1, *w*/*w*/*w*; p.o.). Group 1 received the formulation via tail vein injection, while Groups 2–5 were suspended in water (pH 7.0) and administered orally by gavage at a Cur-equivalent dose of 40 mg/kg. Blood samples (0.2 mL) were collected from each rat at 4, 7, 10, 20, and 30 min, and 1, 2, 4, and 24 h post-dosing. Plasma was separated by centrifugation (5000 rpm, 5 min, 4 °C) and stored at −80 °C until analysis.

Cur was extracted from 50 μL of plasma using 600 μL of ethyl acetate and quantified by LC-MS/MS, with diclofenac sodium (20 μL, 200 ng/mL) serving as the internal standard. Blank plasma was obtained from a separate control group (n = 5) that received water only. Analysis of these blank samples confirmed the absence of significant matrix interference, thereby validating the analytical method.

After dosing, all animals were returned to standard housing conditions as described in Animals section.

#### 2.2.7. Quantitative Determination Methods

##### HPLC/UV-*Vis* Method

Cur content in in vitro dissolution samples was quantified using a Waters 2695 Alliance HPLC system (Waters Corporation, Milford, MA, USA) equipped with a Waters 2998 photodiode array (PDA) detector. Separation was performed on an XBridge^®^ C18 column (4.6 × 250 mm, 5.0 μm; Waters) maintained at 25 °C. The mobile phase, composed of water and methanol (80:20, *v*/*v*), was delivered isocratically at a flow rate of 1.0 mL/min. The injection volume was 10 μL, and detection was performed at 430 nm, and Cur eluted at approximately 4.80 min. The method exhibited linearity over 0.5–1000 μg/mL (R^2^ > 0.999). All data were acquired in triplicate (n = 3) and processed using Empower^®^3 Software (Waters).

##### LC-MS/MS Method

Cur quantification in cellular and rat plasma samples was carried out using a SCIEX QTRAP^®^ 5500 LC-MS/MS system coupled with a SCIEX ExionLC^TM^ AC system (AB SCIEX, Framingham, MA, USA).

Separation was achieved on an Acquity UPLC^®^ BEH C18 column (100 × 2.1 mm, 1.7 μm; Waters) maintained at 40 °C. The mobile phase consisted of (A) 0.1% formic acid in water and (B) acetonitrile (ACN), delivered at a flow rate of 0.14 mL/min with the following gradient program: 10% B (0–1.0 min), increased to 95% B (1.0–9.0 min), and returned to 10% B (9.0–12.0 min). The injection volume was 5 μL. The retention times were approximately 4.85 min for Cur and 6.70 min for diclofenac sodium, used as the internal standard (IS).

Mass spectrometric detection was performed using an IonDrive^TM^ Turbo V ion (SCIEX, Framingham, MA, USA) source operating in negative electrospray ionization (ESI) mode. Quantification was conducted via multiple reaction monitoring (MRM) at *m*/*z* 367.1→133.9 for Cur, and *m*/*z* 294.1→250.0 for diclofenac sodium (IS). The assay was linear over the concentration range of 2-500 ng/mL (R^2^ > 0.999). All data were acquired in triplicate (n = 3) and processed using MultiQuant software version 3.0.2 (AB SCIEX).

All analytical methods underwent comprehensive validation according to standard guidelines for key parameters.

#### 2.2.8. Data Analysis

Pharmacokinetic parameters were calculated using non-compartmental analysis with DAS 2.0 software (Boying Corporation, Beijing, China). Data analysis was performed using GraphPad Prism 10.1.2 software. The result is expressed as mean ± standard deviation (SD). For pairwise comparisons between groups, t-tests were conducted. For multiple group comparisons, one-way analysis of variance (ANOVA) was performed, followed by Tukey’s post hoc test for multiple comparisons. Prior to ANOVA, the assumptions of normality and homogeneity of variances were verified using the Shapiro–Wilk test and Bartlett’s test, respectively. *p* < 0.05 indicates statistically significant differences.

## 3. Results

### 3.1. Drug–Polymer–Surfactant Interactions

#### 3.1.1. Monitoring Intermolecular Interactions by NMR Spectroscopy

^1^H NMR spectroscopy provides atomic-scale insights into intermolecular interactions. We examined these interactions in the solution state using CDCl_3_ as the solvent, chosen for its excellent dissolution capability and minimal interference with hydrogen bonding.

**Figure 2:**
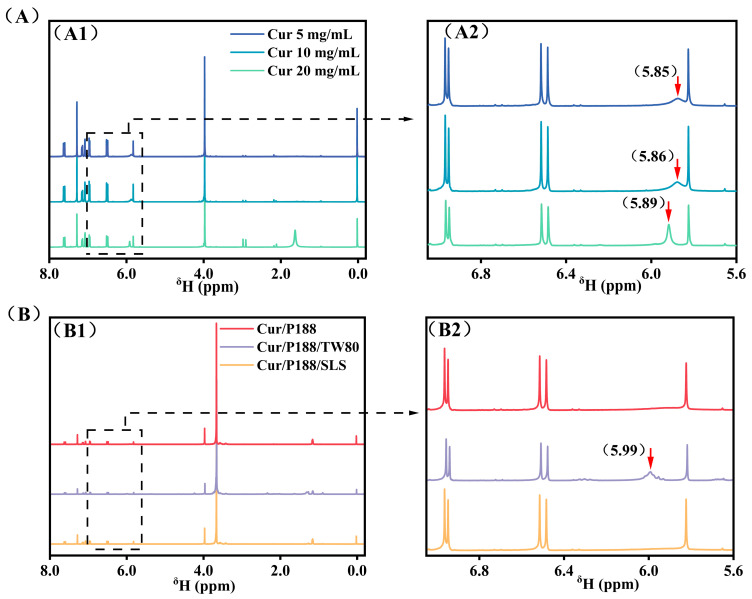
^1^H NMR spectra (400 MHz, CDCl_3_). (**A**) Pure Curcumin (Cur) at 5, 10, and 20 mg/mL. (**B**) Combinations of Cur/P188, Cur/P188/TW80, and Cur/P188/SLS, with each component at 5 mg/mL. Panels (**A1**,**B1**) display the full spectral range (0.0–8.0 ppm), while panels (**A2**,**B2**) show expanded views of the 5.6–7.0 ppm region. Cur phenolic hydroxyl (-OH) signals are indicated by red arrows. Tetramethylsilane (TMS) was used as an internal reference.

Figure 2A compares the ^1^H NMR spectra of pure Cur at concentrations of 5, 10, and 20 mg/mL. The signal corresponding to the hydroxyl (-OH) proton initially at δ 5.85 ppm (5 mg/mL, blue line) shifted downfield to δ 5.86 ppm (10 mg/mL, cyan line) and further to δ 5.89 ppm (20 mg/mL, green line) with increasing concentration. Concurrently, this peak sharpened noticeably (indicated by red arrows), while other proton signals of Cur remained unchanged. This downfield shift accompanied by line-sharpening suggests restricted proton exchange kinetics due to enhanced intermolecular hydrogen bonding among Cur molecules.

Figure 2B compares the ^1^H NMR spectra of the binary Cur/P188 mixture (1:1, *w*/*w*) and the ternary Cur/P188/TW80 and Cur/P188/SLS mixtures (1:1:1, *w*/*w*/*w*), with each component at 5 mg/mL. In the Cur/P188 system, the Cur -OH signal was completely absent (red line), indicating effective encapsulation of Cur by P188 through strong intermolecular hydrogen bonding between Cur -OH groups and the polyoxyethylene (PEO) segments of P188, which shields the proton and restricts its mobility.

The surfactants TW80 and SLS exhibited clearly different influences on Cur-P188 interactions. In the Cur/P188/TW80 system, the Cur -OH signal reappeared at δ 5.99 ppm (purple line), indicating that TW80 interferes with Cur-P188 binding. As a non-ionic surfactant, TW80 also contains PEO units that compete with those of P188 for hydrogen bonding with Cur -OH groups, thereby disrupting the original Cur-P188 interactions and restoring the -OH NMR signal.

In contrast, the Cur/P188/SLS system showed no detectable -OH signal (orange line), similar to the binary system. As an anionic surfactant, SLS functions via a different mechanism: its charged head group participates only in weak dipole–ion interactions with the PEO segments of P188, which are insufficient to compete with Cur for hydrogen bonding sites. More importantly, SLS incorporates into the Cur/P188 nanostructure, acting as a structural stabilizer that reinforces the assembly. As later confirmed by DLS and TEM, the Cur/P188/SLS system forms larger and more stable nanostructures, resulting in persistent shielding of the Cur -OH proton.

In summary, TW80 acts as a competitor, disrupting Cur-P188 binding and likely forming Cur-TW80 interactions, whereas SLS serves as a collaborator, forming a Cur/P188/SLS nanostructure that stabilizes the assembly through complementary integration, thereby enhancing proton shielding and overall system performance.

#### 3.1.2. Monitoring Intermolecular Interactions by FT-IR Spectroscopy

**Figure 3:**
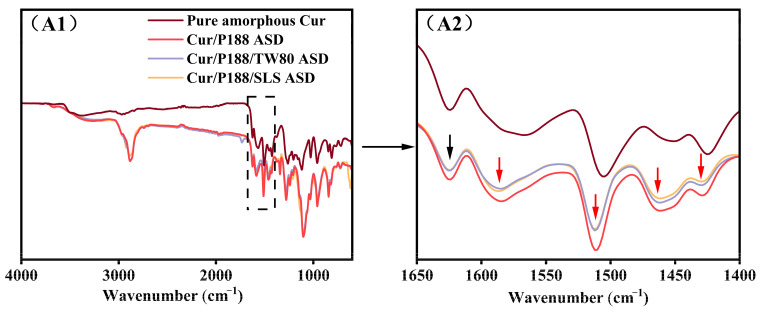
FT-IR spectra (from top to bottom): pure amorphous Cur, Cur/P188 ASD, Cur/P188/TW80 ASD, and Cur/P188/SLS ASD. (**A1**) Full spectra (4000–600 cm^−1^); (**A2**) expanded view of the carbonyl and aromatic region (1650–1400 cm^−1^). Black arrows indicate peaks consistent across pure amorphous Cur and all ASDs; red arrows highlight spectral changes specific to the ASDs relative to pure amorphous Cur.

FT-IR spectroscopy was employed to characterize intermolecular interactions in the solid state. Spectra of pure amorphous Cur, binary Cur/P188 ASD (1:2, *w*/*w*), and ternary Cur/P188/TW80 and Cur/P188/SLS ASDs (1:2:1, *w*/*w*/*w*) were compared, with particular emphasis on the carbonyl and aromatic region (1650–1400 cm^−1^), as shown in Figure 3.

Spectral assignments were referenced to the literature [19]. The spectrum of pure amorphous curcumin (brown line) displayed a characteristic peak at 1624 cm^−1^ and a broad band near 1566 cm^−1^, both assigned to carbonyl (C=O) stretching vibrations. Several aromatic ring vibrations were also observed between 1530 and 1400 cm^−1^, including a peak at 1506 cm^−1^ (C=C stretching), a peak at 1425 cm^−1^, and a broad band around 1450 cm^−1^ (attributed to olefinic C-H bending).

Notable spectral changes were observed in the binary Cur/P188 ASD (red line) compared with pure amorphous Cur. In the carbonyl region, the peak at 1624 cm^−1^ remained unchanged (marked with black arrows), while the broad band originally at 1566 cm^−1^ underwent a blue shift to 1585 cm^−1^ (Δν = +19 cm^−1^; red arrow). In the aromatic region, the peak at 1506 cm^−1^ shifted to 1512 cm^−1^ (Δν = +6 cm^−1^), the broad band at 1450 cm^−1^ shifted to 1463 cm^−1^ (Δν = +13 cm^−1^), and the peak at 1425 cm^−1^ shifted to 1429 cm^−1^ (Δν = +4 cm^−1^); all shifted bands are indicated by red arrows. These spectral changes suggest the disruption of hydrogen bonding and π–π stacking interactions among Cur molecules in the ASD system.

The ternary Cur/P188/TW80 (purple line) and Cur/P188/SLS (orange line) ASDs exhibited nearly identical spectral features in both the carbonyl and aromatic regions to the binary Cur/P188 ASD. This indicates that the incorporation of either TW80 or SLS does not disrupt the solid-state interactions between Cur and P188.

The FT-IR results suggest that Cur molecules undergo self-association through hydrogen bonding and π–π stacking, which aligns with previous reports on its molecular structure and associated intermolecular behavior [20]. Such self-interaction, mediated by functional groups such as phenolic hydroxyls and phenyl rings, is presumed to contribute to Cur’s inherent crystallization tendency and poor aqueous solubility. As demonstrated in Section 3.2, the introduction of polymer and surfactants in the ASD system effectively disrupts these intermolecular interactions, thereby facilitating the enhanced dissolution of Cur in aqueous media.

### 3.2. In Vitro Dissolution of Cur Formulations

#### 3.2.1. Solubility of Crystalline Cur in Excipient Solutions

**Figure 4:**
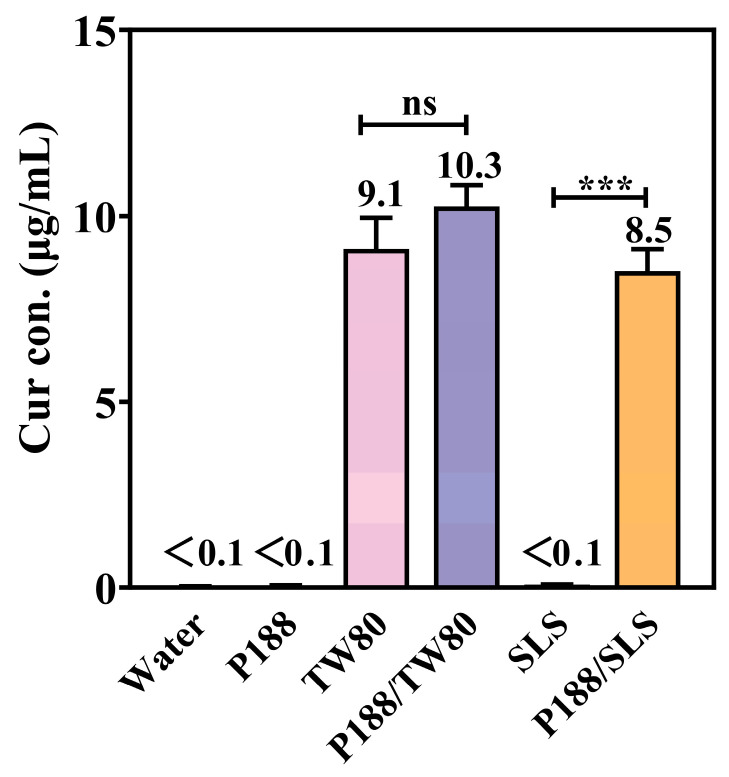
Equilibrium solubility of crystalline Cur in aqueous solutions containing P188, TW80, SLS, or their combinations (P188/TW80 and P188/SLS) (Mean ± SD, n = 4). ns, not significant; *** *p* < 0.001 for individual surfactants compared to the corresponding polymer/surfactant combinations (Student’s *t*-test).

The equilibrium solubility of crystalline Cur was determined in aqueous solutions containing individual components of P188 (8 mg/mL), TW80 (1 mg/mL), or SLS (1 mg/mL), and their combinations (P188/TW80 or P188/SLS, 8:1 *w*/*w*; 8 mg/mL + 1 mg/mL). The results are summarized in Figure 4.

Crystalline Cur exhibited negligible intrinsic solubility in water (<0.1 μg/mL). Neither P188 nor SLS alone improved Cur solubility, which remained below 0.1 μg/mL. In contrast, TW80 alone significantly increased solubility to 9.1 μg/mL, likely due to micellar solubilization. The P188/TW80 combination showed no synergy, with solubility (10.3 μg/mL) comparable to TW80 alone. Conversely, the P188/SLS combination showed a clear synergistic enhancement, increasing solubility to 8.5 μg/mL, which is significantly higher (*p* < 0.001) than either component alone (<0.1 μg/mL). These results indicate that P188 and SLS act synergistically to improve Cur solubility, while no such cooperative effect was observed between P188 and TW80.

#### 3.2.2. Dissolution Performance

The dissolution behavior of Cur formulations was evaluated under non-sink conditions at a Cur-equivalent concentration of 1 mg/mL, significantly exceeding its crystalline solubility (<0.1 μg/mL). The study systematically assessed (1) dissolution kinetics; (2) physicochemical properties of drug nanostructures formed during dissolution; and (3) crystallinity of precipitated drug. Pure amorphous Cur was compared with binary Cur/P188 ASD (1:8, *w*/*w*) and ternary Cur/P188/TW80 and Cur/P188/SLS ASDs (1:8:1, *w*/*w*/*w*).

**Figure 5:**
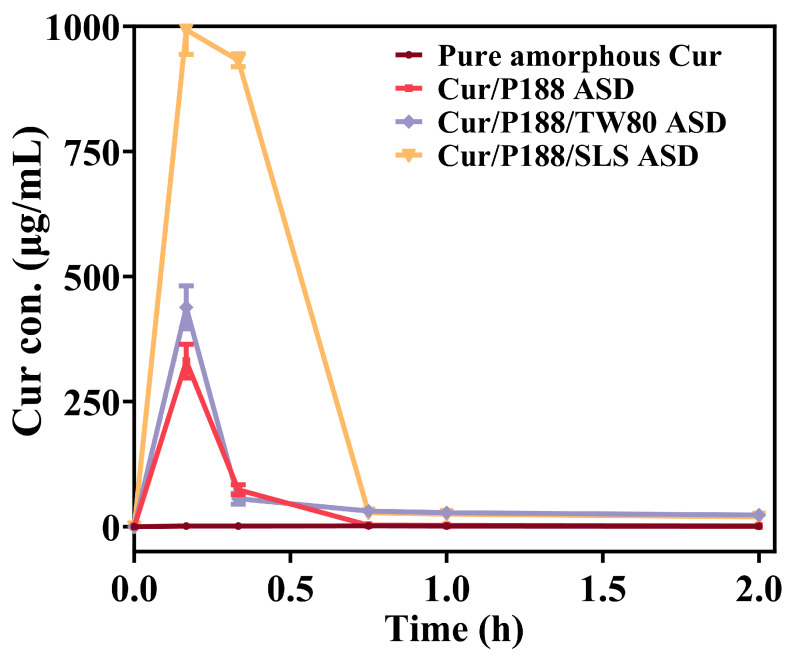
In vitro dissolution profiles of pure amorphous Cur and Cur ASDs (Cur/P188, Cur/P188/TW80, and Cur/P188/SLS) under non-sink conditions using a Cur-equivalent concentration of 1 mg/mL (Mean ± SD, n = 3).

As shown in Figure 5, pure amorphous Cur (brown line) rapidly crystallized upon hydration, reaching only ~1 μg/mL over 2 h. The binary Cur/P188 ASD (red line) showed improved yet slow and incomplete dissolution: the concentration peaked at 330.9 μg/mL at 10 min, then declined rapidly to 73.7 μg/mL (20 min) and further to 1.5 μg/mL (2 h).

The ternary ASDs showed distinct dissolution profiles. The Cur/P188/TW80 ASD (purple line) performed similarly to the binary ASD, peaking at 438.4 μg/mL (10 min) before dropping to 55.8 μg/mL (20 min) and stabilizing at 23.2 μg/mL (2 h). Its moderately higher concentrations after 45 min may be attributed to the solubilizing effect of TW80. In contrast, the Cur/P188/SLS ASD (orange line) demonstrated markedly enhanced performance, achieving near-complete dissolution (993.0 μg/mL) within 10 min. However, the resulting supersaturation formed a metastable state that persisted for only about 20 min (932.1 μg/mL), after which the drug concentration decreased sharply to 28.0 μg/mL (45 min) and further to 19.5 μg/mL (2 h).

**Figure 6:**
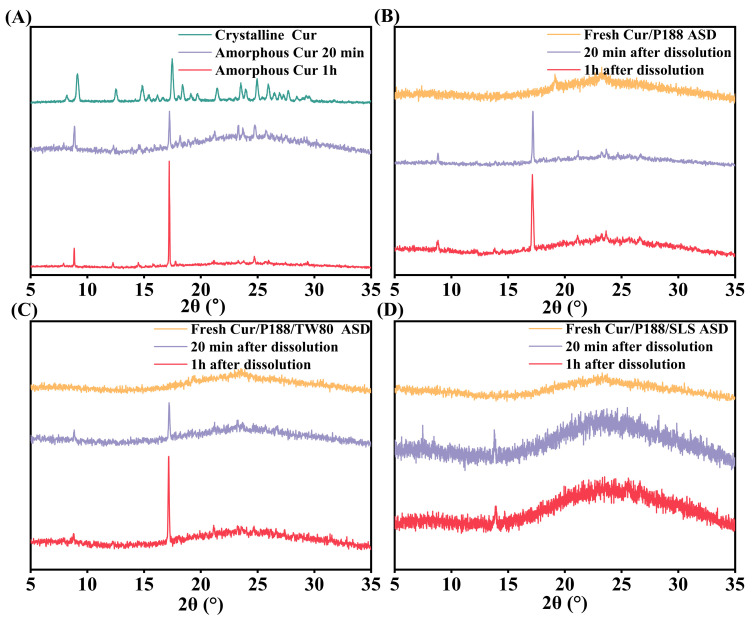
XRD patterns of Cur formulations before and after dissolution: (**A**) amorphous Cur; (**B**) Cur/P188 ASD; (**C**) Cur/P188/TW80 ASD; (**D**) Cur/P188/SLS ASD. Precipitates were collected at 20 min and 1 h. Crystalline Cur is included as a reference.

Figure 6 presents the XRD patterns of freshly prepared Cur materials and corresponding precipitates collected after 20 min and 1 h of dissolution. Crystalline Cur exhibited sharp, high-intensity diffraction peaks, confirming its ordered structure (Figure 6A, green line). Pure amorphous Cur crystallized immediately upon hydration, with precipitates at both time points showing characteristic crystalline peaks at 17.2° (2θ) (Figure 6A, purple and red lines), matching the reference pattern of crystalline Cur. Figure 6B–D present the results for Cur/P188, Cur/P188/TW80, and Cur/P188/SLS ASDs, respectively. All freshly prepared ASDs showed halo patterns, confirming their amorphous nature (orange lines in Figure 6B–D). Precipitates from the binary Cur/P188 ASD exhibited emerging crystalline peaks at 17.2° (2θ), with intensity increasing over time, indicating progressive crystallization during dissolution (Figure 6B, purple and red lines).

The ternary ASDs showed divergent crystallization behaviors: precipitates from the Cur/P188/TW80 ASD also exhibited distinct crystalline peaks at 17.2° (2θ) (Figure 6C, purple and red lines), confirming that TW80 did not inhibit crystallization. In contrast, precipitates from the Cur/P188/SLS ASD maintained amorphous halos with no crystalline peaks at either time point (Figure 6D, purple and red lines), demonstrating the effective crystallization inhibition by SLS.

**Figure 7:**
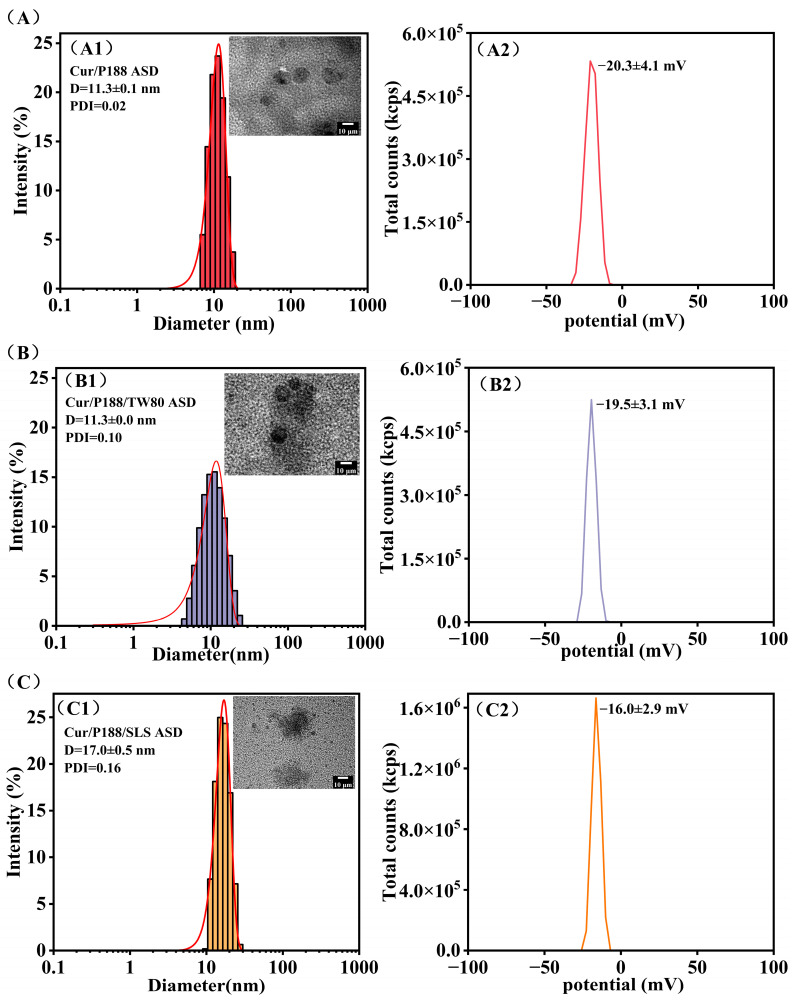
Characterization of the nanostructures formed during the dissolution of Cur ASDs: (**A**) Cur/P188 ASD, (**B**) Cur/P188/TW80 ASD, and (**C**) Cur/P188/SLS ASD. For each formulation, the hydrodynamic size distribution (DLS) and a representative TEM image (inset) are shown in panels (**A1**–**C1**), respectively; the corresponding zeta potential is presented in panels (**A2**–**C2**) (n = 3).

Figure 7 characterizes the drug nanostructures formed during ASD dissolution using DLS and TEM (insets). All Cur ASDs generated small, uniform, spherical nanostructures with low polydispersity indices (PDIs), indicating homogeneous size distributions. The Cur/P188 and Cur/P188/TW80 ASDs produced nanostructures with similar z-average diameters (11.3 nm; Figure 7(A1,B1)) and zeta potentials −20.0 mV; Figure 7(A2,B2)). This similarity aligns with the preceding NMR analysis (Section 3.1.1), which indicated poor compatibility of TW80 with the Cur/P188 system and a lack of effective integration into the nanostructures. In contrast, the Cur/P188/SLS ASD generated nanostructures with a larger z-average diameter (17.0 nm; Figure 7(C1)) and a less negative zeta potential −16.0 mV; Figure 7(C2)); this difference is attributed to the compatibility of SLS and its molecular interactions with Cur/P188, as shown by NMR analysis), enabling its incorporation into a more stable ternary assembly.

Zeta potential values provide critical insight into the electrostatic stabilization of these nanostructures. The more negative zeta potentials (−20 mV) of nanostructures formed in Cur/P188 and Cur/P188/TW80 systems are likely dominated by the inherent properties of PEO segments on P188 or TW80 [21]. The moderately negative zeta potential of the Cur/P188/SLS nanostructures (−16 mV) originates from the incorporation of anionic SLS, whose sulfate head groups are presented at the particle–solution interface. Although a higher absolute zeta potential can indicate good stability, the successful integration of SLS to form more stable Cur/P188/SLS nanostructures contributes to enhanced drug solubilization, crystallization inhibition, and membrane transport (Section 3.3.2).

In summary, dissolution studies revealed that the rapid precipitation and poor supersaturation of pure amorphous Cur are consistent with classical nucleation theory, where the high free energy of the amorphous form drives recrystallization in the absence of effective inhibitors [22]. Cur ASDs significantly enhanced dissolution performance by disrupting Cur self-interaction and serving as effective crystallization inhibitors, while also forming nanostructures that improve Cur solubilization. However, dissolution performance varied considerably among the ASDs, which can be attributed to the distinct roles of surfactants in modulating drug–polymer interactions and nanostructure formation during dissolution [23].

The binary Cur/P188 ASD and ternary Cur/P188/TW80 ASD showed similar yet limited dissolution, likely due to the compromised solubilizing capacity of P188 and TW80, which temporarily sequesters drug molecules but fails to provide long-term supersaturation stabilization or effective crystal growth inhibition. Due to their similar PEO-containing structures, TW80 did not exhibit a synergistic effect with P188 to enhance solubilization or prevent crystallization of amorphous Cur.

In contrast, the Cur/P188/SLS ASD exhibited superior performance, including rapid generation and prolonged maintenance of high supersaturation, along with effective inhibition of precipitate crystallization. These properties collectively maintained a greater proportion of drug molecules in high-energy states, considering both those in the supersaturated solution and those within amorphous precipitates. This enhancement likely results from multiple factors: (1) the anionic nature of SLS provides electrostatic stabilization to the nano-assemblies; (2) its specific interaction with the Cur-P188 complex, without disrupting the primary drug–polymer binding, but facilitates the formation of more stable Cur/P188/SLS nanostructures, as evidenced by zeta potential and DLS/TEM results, which help prolong supersaturation; and (3) SLS can adsorb onto nascent crystal surfaces, thereby blocking active growth sites [24].

### 3.3. Cellular Uptake of Cur Formulations

#### 3.3.1. Cytotoxicity in MDCK Cells

**Figure 8:**
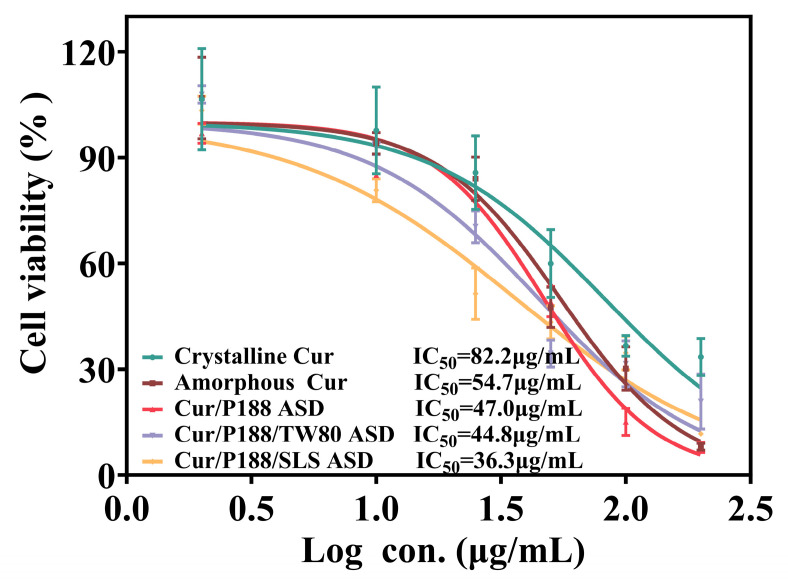
Viability of MDCK cells after 4 h of treatment with crystalline Cur, amorphous Cur, and Cur ASDs (Cur/P188, Cur/P188/TW80, and Cur/P188/SLS) (Mean ± SD, n = 5).

The cytotoxicity of various Cur formulations was evaluated using a cell viability assay (Figure 8). Crystalline Cur showed negligible toxicity at concentrations ≤25 μg/mL but exhibited concentration-dependent cytotoxicity at higher concentrations, with an IC_50_ value of 82.2 μg/mL, consistent with literature reports in Caco-2 cells [25,26]. All Cur formulations demonstrated greater cytotoxicity than crystalline Cur, with Cur-equivalent IC_50_ values as follows: 54.7 μg/mL (amorphous Cur), 47.0 μg/mL (Cur/P188 ASD), 44.8 μg/mL (Cur/P188/TW80 ASD), and 36.3 μg/mL (Cur/P188/SLS ASD). At a Cur-equivalent concentration of 10 μg/mL, all formulations maintained cell viability above 80% after 4 h of exposure, indicating negligible cytotoxicity and establishing their suitability for subsequent cellular uptake studies.

#### 3.3.2. Cellular Uptake of Cur by MDCK Cells

**Figure 9:**
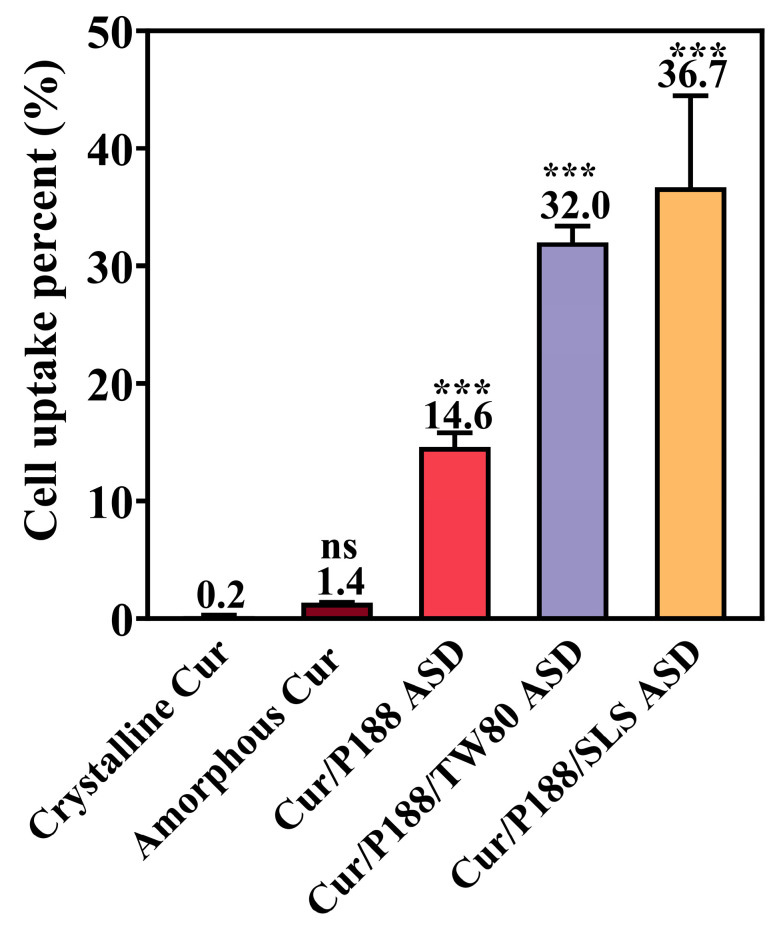
Cellular uptake of crystalline Cur, amorphous Cur, and Cur ASDs (Cur/P188, Cur/P188/TW80, and Cur/P188/SLS) by MDCK cells after 10 min incubation at a dose of 5 μg Cur-equivalent (Mean ± SD, n =4). ns, not significant; *** *p* < 0.001 compared to crystalline Cur (one-way ANOVA).

Cellular uptake was quantified after treating MDCK cells (cultured in 24-well plates) with 500 µL of complete DMEM containing each Cur formulation at a Cur-equivalent concentration of 10 μg/mL (5 µg total dose) (Figure 9). Crystalline Cur showed negligible cellular uptake (0.2% of the administered dose), while amorphous Cur showed a moderate increase (1.4%). All three ASD formulations significantly enhanced uptake: the binary Cur/P188 ASD reached 14.6%, and the ternary ASDs of Cur/P188/TW80 and Cur/P188/SLS further increased uptake to 32.0% and 36.7%, respectively.

The enhanced cellular uptake observed with the ASD formulations is not attributable to cytotoxicity, as all tested amounts maintained high cell viability in earlier cytotoxicity assays (Section 3.3.1). The improvement likely stems from increased drug solubility and may involve nanoparticle-facilitated transport pathways, such as endocytosis. Moreover, the surfactant-containing ternary ASDs exhibited the highest cellular uptake, possibly due to surfactant-induced increases in cell membrane permeability. Further studies are required to confirm this hypothesis.

### 3.4. Pharmacokinetics of Cur Formulations

**Figure 10:**
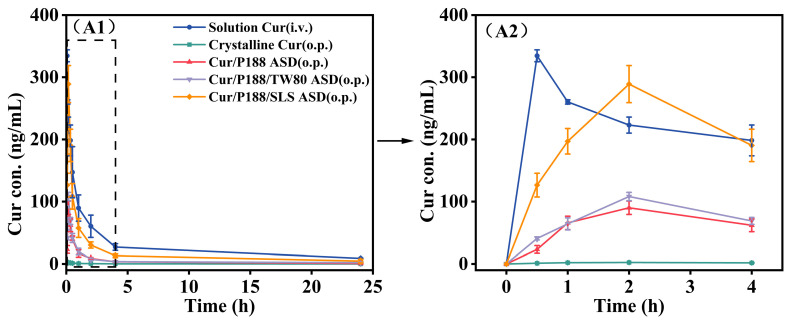
Mean plasma concentration–time profiles of Cur in rats following oral or intravenous administration of various Cur formulations at a Cur-equivalent dose of 40 mg/kg. (**A1**) Full profiles (0–24 h); (**A2**) expanded view of the initial 4 h phase. (Mean ± SD, n = 5).

The oral bioavailability of various Cur formulations was evaluated in Sprague-Dawley (SD) rats at a Cur-equivalent dose of 40 mg/kg. Plasma concentration–time profiles (Figure 10) and pharmacokinetic parameters derived from a two-compartment model are summarized in Table 1.

Crystalline Cur exhibited poor gastrointestinal absorption, with an absolute oral bioavailability of only ~1.0% relative to intravenous administration (Cur dissolved in saline containing 5% DMSO), which is consistent with literature-reported values [27]. Notably, all Cur ASDs achieved significantly higher maximum plasma concentrations (C_max_, *p* < 0.001) and area under the curve (AUC_0–24h_, *p* < 0.001) values compared to crystalline Cur, resulting in substantially enhanced absolute bioavailability: 15.7% for Cur/P188 ASD, 18.6% for Cur/P188/TW80 ASD, and 56.2% for Cur/P188/SLS ASD. This improvement is primarily attributed to the amorphous nature of Cur in the ASD formulations, which enhances dissolution, generates supersaturated drug concentrations, and promotes the formation of nanostructures that facilitate transport across the gastrointestinal membrane.

Among the ASD formulations, Cur/P188 ASD and Cur/P188/TW80 ASD showed comparable in vivo performance, with no significant differences in C_max_ or AUC_0–24h_ (*p* > 0.05), indicating that the addition of TW80 did not significantly enhance oral bioavailability. This observation aligns with in vitro dissolution results, which demonstrated that TW80 neither improved dissolution nor inhibited crystallization. In contrast, the Cur/P188/SLS ASD significantly outperformed all other ASD formulations (*p* < 0.001), achieving the highest bioavailability. This superior performance can be attributed to the ability of this ASD to (1) enhance and sustain drug supersaturation; (2) effectively inhibit the crystallization of precipitates; and (3) promote the formation of nanostructures that improve epithelial permeability and cellular uptake, thereby facilitating efficient transepithelial absorption.

## 4. Discussion

Cur was selected as a model compound in this study due to its extremely low oral bioavailability, resulting from poor aqueous solubility and chemical instability, which greatly restrict its clinical utility. Numerous studies have employed ASD technology to enhance the dissolution and bioavailability of Cur. Hydrophilic polymers such as hydroxypropyl methyl cellulose (HPMC) and polyvinylpyrrolidone (PVP) have proven effective as carriers for Cur ASDs [28]. The combination of polymer carriers, such as HPMC with sodium carboxymethyl cellulose (CMC), has also been demonstrated to enhance anti-proliferative efficacy and bioavailability [29]. In addition, co-delivering Cur with other bioactive compounds (e.g., piperine or resveratrol) to form ternary drug–drug–polymer ASDs represents a promising strategy that markedly improves dissolution and bioaccessibility [30,31]. Surfactants (non-ionic, anionic, and cationic) can further overcome Cur’s limitations through micellar solubilization [32]. The combined use of polymers and surfactants as co-carriers leverages the polymer’s ability to stabilize the drug in the amorphous state and the surfactant’s micellar effect, substantially enhancing both in vitro and in vivo performance [33,34]. These studies highlight that rational ASD design through appropriate polymer selection, drug co-delivery, polymer combinations, or polymer–surfactant blends, can significantly improve the bioavailability of Cur. In particular, surfactants display a promising role; however, the mechanisms underlying their bioavailability-enhancing effects remain inadequately elucidated, hindering rational surfactant selection in ASD design. This study aims to address this issue by systematically evaluating how structurally diverse surfactants influence the dissolution and in vivo bioavailability of Cur ASDs and explored in vitro–in vivo correlations (IVIVCs) to guide formulation design.

Surfactants can influence critical pharmaceutical behaviors of ASD by intervening in multiple interrelated dissolution processes. Upon aqueous exposure, surfactants enhance wettability and dissolution rate, facilitating the generation of a highly supersaturated drug solution. Although thermodynamically unstable and prone to precipitation and crystallization, surfactants can stabilize drug supersaturation through micellization or nanostructure formation, which not only inhibits precipitation and crystallization but also promotes gastrointestinal drug absorption [7,32]. These in vitro processes are interdependent and closely linked to in vivo bioavailability. In this study, to gain a comprehensive mechanistic understanding of how surfactants influence Cur ASD performance, we investigated (i) the effects of surfactants on dissolution performance, including dissolution kinetics, supersaturation generation and maintenance, and crystallization inhibition; (ii) their influence on nanostructure formation and cellular drug uptake; and (iii) the molecular interactions among drug, polymer, and surfactant that govern dissolution, absorption, bioavailability, and IVIVC.

Interactions between the drug and polymer are critical for ASD performance. Non-covalent interactions, such as hydrogen bonding and hydrophobic effects, play key roles in enhancing physical stability, drug release, and bioavailability [35]. Surfactants, by virtue of their amphiphilic nature, inherently modulate these interactions and influence dissolution behavior [7,36]. In this study, Cur molecules were observed to participate in hydrogen bonding and π-π stacking, which contribute to their limited aqueous solubility. The polymer P188 disrupts these drug–drug interactions and promotes the formation of more stable drug–polymer associations. The tested surfactants distinctly affected Cur-P188 interactions: TW80 competitively interfered with intermolecular bonding between Cur and P188 and instead formed Cur-TW80 interactions in solution, whereas SLS did not interfere with Cur-P188 binding but likely formed a Cur-P188-SLS interaction network, thereby reinforcing the assembly. These divergent ternary interactions profoundly influenced the in vitro dissolution, cellular uptake, and in vivo bioavailability of Cur ASDs, as well as their intercorrelations.

An optimal dissolution profile for ASD requires both high supersaturation and prolonged drug concentration maintenance. However, elevated supersaturation increases the risk of precipitation, and undissolved amorphous particles may crystallize over time, thereby diminishing the kinetic solubility advantage of the amorphous form. Thus, surfactant selection must carefully balance supersaturation generation with crystallization inhibition. In this study, the binary Cur/P188 ASD showed moderate enhancement in dissolution and supersaturation generation, but exhibited limited capacity to maintain the drug in high-energy states (supersaturated solution or amorphous precipitates). The surfactants exerted distinct effects on dissolution performance through their differential modulation of drug–polymer interactions. Specifically, in the ternary Cur/P188/TW80 system, TW80 showed poor compatibility with the Cur/P188 assembly. Due to its structural similarity to P188 and lack of synergistic interaction, the Cur/P188/TW80 ASD displayed incomplete dissolution and early crystallization, mirroring the behavior of the binary system. In contrast, the ternary Cur/P188/SLS ASD achieved complete dissolution, high supersaturation generation and maintenance, and effective crystallization inhibition. This superior performance can be attributed to the molecular compatibility of SLS with both Cur and P188, which enabled its incorporation into the nanostructure through a specific interaction network. As a result, the Cur/P188/SLS ASD exhibited rapid and complete dissolution, sustained supersaturation, and amorphous precipitates that remained stable for over one hour.

ASD dissolution produces multiple high-energy drug species, including free molecules, nanoscale aggregates, and amorphous precipitates, all of which critically influence bioavailability [37]. Free drug molecules facilitate passive membrane diffusion, while nanoscale structures (<100 nm) function as dynamic drug reservoirs that rapidly replenish drug concentrations absorbed across the intestinal epithelium [38]. These nanostructures may also promote endocytic uptake. The physical state of precipitates is equally important: amorphous aggregates can serve as dissolving reservoirs, whereas crystalline forms lose the amorphous advantage and lead to poor absorption. In this study, DLS and TEM analyses confirmed that all three Cur ASDs formed small, uniform, and spherical nanostructures. Due to its incompatibility with the Cur-P188 assembly, TW80 was not effectively incorporated, resulting in nanostructures with similar size and zeta potential to those of the binary Cur/P188 system. In contrast, SLS was successfully integrated into the Cur-P188 assembly, leading to the formation of Cur/P188/SLS nanostructures with larger particle size and a lower zeta potential.

Cellular uptake studies indicated that crystalline Cur was poorly absorbed. Amorphous Cur showed improved uptake, attributable to its higher-energy state facilitating membrane permeation, although overall absorption remained limited. The Cur/P188 ASD further increased uptake, likely due to improved dissolution and supersaturation, as well as nanostructure-facilitated cellular uptake. The incorporation of either TW80 or SLS markedly enhanced uptake compared to the binary ASD, possibly through surfactant-induced increases in membrane permeability or activation of membrane transport mechanisms. Among all formulations, the Cur/P188/SLS ASD demonstrated the highest cellular uptake, potentially driven by SLS-mediated enhancement of membrane permeability; however, the underlying mechanisms require further systematic investigation.

The in vitro dissolution and cellular uptake trends correlated directly with in vivo bioavailability outcomes. Crystalline Cur exhibited negligible absorption and very low bioavailability. Both the Cur/P188 and Cur/P188/TW80 ASDs provided moderate and comparable bioavailability, indicating a negligible effect of TW80. In contrast, the Cur/P188/SLS ASD significantly enhanced bioavailability, demonstrating the ability of SLS to promote oral absorption. These results confirm that the influence of surfactants correlates with their modulation of key dissolution processes, including improved wettability to enhance dissolution, and the generation and maintain supersaturation via nanostructures, and inhibition of crystallization. Furthermore, increased free drug concentration and nanostructure formation contributed to enhanced cellular uptake and epithelial permeation, collectively leading to superior bioavailability.

This study demonstrates that surfactant selection critically influences ASD performance, with the ternary Cur/P188/SLS system exhibiting superior bioavailability. However, the direct translation of these findings requires careful consideration of species-specific differences. The pharmacokinetic parameters and bioavailability observed in Sprague-Dawley rats may not directly extrapolate to humans due to interspecies variations in gastrointestinal physiology, metabolic pathways, and transporter expression profiles. Furthermore, the use of healthy male animals does not account for potential modifications in drug absorption and disposition under disease conditions.

While these results provide strong mechanistic insights and justify further formulation optimization, additional validation in more complex and human-relevant models is necessary to establish clinical predictive value and support translational development.

## 5. Conclusions

This study provides the first comprehensive mechanistic understanding of how surfactants affect the oral bioavailability of Cur ASDs. We have demonstrated that surfactants modulate ASD dissolution and bioavailability through multifaceted mechanisms governed by drug–polymer–surfactant interactions. While curcumin (Cur) molecules tend to self-associate through intermolecular interactions that limit aqueous dissolution, formulating Cur as an ASD disrupts these drug–drug interactions and establishes more favorable drug–polymer associations. The incorporation of a synergistic surfactant such as SLS further enhances ASD wetting and dissolution, leading to high and sustained supersaturation. The resulting drug/polymer/surfactant nanostructures help maintain supersaturation, stabilize amorphous precipitates, and promote transepithelial drug uptake, collectively improving bioavailability. In contrast, poorly compatible surfactants like TW80 fail to integrate effectively into the nanostructure, offering limited dissolution or absorption benefits.

These findings address the critical challenge of rational surfactant selection in ASD design. We have established that successful surfactant incorporation requires molecular compatibility with both drug and polymer, as exemplified by SLS, which reinforces the ternary assembly without disrupting primary drug–polymer interactions. By combining multi-technique characterization of intermolecular interactions with a multidimensional analysis of dissolution behavior, including nanostructure formation and precipitate stability, this work provides a rational framework for selecting surfactants in ASD formulations and identifies key in vitro performance markers predictive of in vivo bioavailability.

## Figures and Tables

**Table 1:** Pharmacokinetic parameters of Cur in rats following oral or intravenous administration of various Cur formulations at a Cur-equivalent dose of 40 mg/kg, based on a two-compartment model (Mean ± SD, n = 5).

Parameters	Solution Cur (i.v.)	Crystalline Cur (o.p.)	Cur/P188 ASD (o.p.)	Cur/P188/TW80 ASD (o.p.)	Cur/P188/SLS ASD (o.p.)
AUC_(0–24)_/h	697.8 ± 78.4	6.8 ± 1.9	109.3 ± 15.7 ^###^	129.8 ± 13.1 ^###, ns^	392.3 ± 56.2 ^###,^ ***
C_max_/(ng/mL)	334.5 ± 9.6	2.4 ± 0.4	90.1 ± 10.8 ^###^	108.2 ± 6.7 ^###, ns^	289.0 ± 29.8 ^###,^ ***
MRT_(0–24)_/h	5.2 ± 0.2	6.3 ± 2.0	3.6 ± 1.1	5.6 ± 0.7	4.6 ± 1.4
T_max_/h	0.1 ± 0.0	0.2 ± 0.0	0.2 ± 0.0	0.2 ± 0.0	0.2 ± 0.0
T_1/2_/h	1.8 ± 0.2	1.8 ± 0.4	1.9 ± 0.9	1.2 ± 0.2	1.4 ± 0.4
F (%)		0.97	15.66	18.60	56.22

**Abbreviations:** AUC: area under the plasma concentration–time curve; C_max_: maximum plasma, concentration; MRT: mean residence time; T_max_: time to reach C_max_; T_1/2_: elimination half-life. **Statistical Significance:** ^ns^, not significant; ^###^ *p* < 0.001 vs. crystalline Cur (o. p.); *** *p* < 0.001 vs. Cur/P188 ASD (one-way ANOVA). (Mean ± SD, n = 5).

## Data Availability

The original contributions presented in this study are included in the article/Supplementary Material. Further inquiries can be directed to the corresponding authors.

## Supplementary Materials

The following supporting information can be downloaded at: https://www.mdpi.com/article/10.3390/pharmaceutics17121541/s1, Plasma concentrations of Cur over time in individual animals after oral administration of different formulations (n = 5).
